# Rotator Cuff Health, Pathology, and Repair in the Perspective of Hyperlipidemia

**DOI:** 10.26502/josm.511500063

**Published:** 2022-10-17

**Authors:** Armand N Yazdani, Vikrant Rai, Devendra K Agrawal

**Affiliations:** Department of Translational Research, College of Osteopathic Medicine of the Pacific, Western University of Health Sciences, Pomona, California, 91766, USA

**Keywords:** Fatty infiltration, Hyperlipidemia, Inflammation, Molecular pathogenesis, Rotator cuff injury

## Abstract

Rotator Cuff Injuries (RCI) are prevalent cause of shoulder pain affecting over 20% of the population in the USA. Surgical repair of the torn rotator cuff helps in relieving the pressure on the rotator cuff tendon and from symptoms, however tendon-to-bone healing after rotator cuff surgery still has a high failure rate. Hyperlipidemia has been strongly associated with RCI although the cellular and molecular mechanisms are largely unknown. The focus of this critical review is to further explore the role of hyperlipidemia in RCI and rotator cuff tissue repair to determine its implication as a risk factor for tears, repair, and retears. A literature review was conducted to elucidate the role of hyperlipidemia as an inflammatory mediator and catalyst for structural instability within the shoulder. The results from various studies were critically reviewed to summarize the relationship between hyperlipidemia and rotator cuff pathology. Hyperlipidemia induces LDL-particle entrapment within the dense regular collagen of rotator cuff tendons resulting in foam cell aggregation and macrophage recruitment. Subsequent inflammatory pathways including the JAK2/STAT3 pathway and NLRP3 inflammasome pathway led to persistent inflammation and Extracellular Matrix (ECM) degradation within the rotator cuff. While arthroscopic repair remains the most common treatment modality, nonsurgical treatment including statins, vitamin D, and targeting miRNA are also of therapeutic benefit. Hyperlipidemia interferes with arthroscopic repairs by inducing inflammation and stiffness within tendons and increases the risk of retears. Most notably, targeting underlying mechanisms influencing inflammation has large therapeutic value as a novel treatment strategy for the management of rotator cuff pathology.

## 1. Introduction

Rotator Cuff tears are a very prevalent cause of shoulder pain; population-based studies suggest that 20.7% of the population has a full-length tear [1,2]. In adults, rotator cuff tears are the most common tendon injury seen and treated [3]. The rotator cuff is constituted of the supraspinatus, subscapularis, infraspinatus, and teres minor muscles [4]. Tendons for these muscles cross the coronal and transverse plane of the shoulder joint allowing for force coupling and large-scale rotational motion however, the tendons are susceptible to impingement and inflammation. When a tear is introduced, the force couples are disrupted and motion within the joint is restricted [5].

Rotator Cuff Injury (RCI) is associated with shoulder pain, change in shoulder movement to avoid pain, decreased range of motion, increased pain and difficulty with overhead activity, pain radiating down into the deltoid muscle area, and muscle atrophy [3,4]. Mainly five risk factors have been strongly associated with rotator cuff tears including age, hand dominance, smoking, hypertension, and body weight [3,6–9]. However, within these studies, there is a call for more focused research on the association between obesity and rotator cuff tears [9]. Hyperlipidemia, related to obesity, is a metabolic disease characterized by imbalanced blood lipid profiles which can cause damage to soft tissues including cartilage and tendons [1]. On a grand scale, hyperlipidemia has progressed linearly with the obesity epidemic in the United States as hyperlipidemia has a strong association with high Body Mass Index (BMI) [10,11]. Studies suggest a strong causal relationship between hyperlipidemia and rotator cuff tears via many molecular mechanisms including inflammation, osteoclast migration, xanthoma accumulation, and extracellular matrix disorganization [12–16]. However, there is still no defined therapeutic target to improve clinical outcomes, and thus, there is a need for an in-depth understanding of the underlying molecular mechanism of RCI in a hyperlipidemic environment.

Common treatment strategies include both non-surgical statin therapies and surgical arthroscopic repair; however, statin therapy is associated with negative effects including muscle rhabdomyolysis [17,18] and decreased tendon thickness, tendinopathy, and tendon rupture [19,20] while the arthroscopic repair is associated with poor tendon-to-bone healing [15,21]. These therapies fail to target the underlying molecular mechanisms of hyperlipidemia-induced inflammation and extracellular matrix degeneration [15,22]. The rate of rotator cuff tears has only increased, therefore, there is a need for better treatment strategies that lessen the role of hyperlipidemia as a causative factor for rotator cuff tears, impaired repair, and retear.

## 2. Hyperlipidemia and Three Models of Tendon Health

The strength of the rotator cuff is largely determined by the tendons of the rotator cuff muscles. Three predominant theories characterize tendon pathologies: the vascular theory describes how tendons, unlike bone, do not have a direct vascular supply which can result in tearing and degeneration. The mechanical theory predicts tendon failure and degeneration because of an inability of the tendon to respond to load. The neural theory highlights the proinflammatory components of tendon degeneration [23]. The effects of hyperlipidemia can be seen in all three models of tendon health. With regards to the vascular theory, hyperlipidemic patients are subject to increased plasma Low-Density Lipoprotein (LDL) components that aggregate in the form of xanthomas inside the tendons in addition to increasing the risk of atherosclerotic cardiovascular disease [1,15,24]. This accumulation directly ties into the neural theory of tendon degeneration as the accumulation of LDL components within the tendon matrix causes chemokine recruitment to the localized area followed by tissue necrosis [25]. Finally, hyperlipidemia also influences the mechanical theory as the hyperlipidemic-induced osteoclast migration at the head of the humerus accompanied by bone mineralization and decrease of tendon insertion strength at the infraspinatus enthesis will ultimately affect the mechanical ability of the rotator cuff to respond to load [26–28].

## 3. Hyperlipidemia, Molecular Mechanisms, and RCI

### 3.1 Hyperlipidemia

In a broad sense, Hyperlipidemia (HL) is a metabolic disorder characterized by elevated blood lipid levels because of genetic disorders, unhealthy diet, medications, hypothyroidism, diabetes, or poor lifestyle regimen [1,25]. Statistically, HL can be referred to as high levels of low-density lipoproteins, cholesterol, or triglycerides greater than 90% of the population [25]. Of the lipoproteins, high triglycerides and low High-Density Lipoprotein (HDL) cholesterol are most associated with worse pain and outcomes of rotator cuff tears [29]. Economically, hyperlipidemia has contributed to the over $195.6 billion cost associated with cardiovascular diseases (CVD) – 61% of total healthcare costs [30]. The polygenic nature of hyperlipidemia makes the treating individual disorders a challenge however environmental factors that influence pathology include obesity, high saturated fat intake, and high cholesterol. Hereditary mechanisms such as elevated Apo B-100 levels can also increase the risk of developing the disorder [25]. Longitudinal population-based studies suggest a clear association between hyperlipidemia and Rotator Cuff Disease (RCD) indicating that out of 25,621 hyperlipidemic patients, 9.7% will go on to develop an RCD [1]. Patients with hyperlipidemia are also found to have more severe rotator cuff tears [31]. On a grand scale, hyperlipidemia has progressed linearly with the obesity epidemic in the United States [11]. As obesity rates continue to rise, hyperlipidemia-focused research becomes more impactful for the wellness of large populations suffering from shoulder pain.

A hyperlipidemic environment at the site of injury can exacerbate the inflammatory response and further cause cholesterol buildup and necrosis of tissues [25]. Recent findings, focused on treatment, reveal the importance of High Mobility Group Box Protein (HMGB)1 and NOD-, LRR- And Pyrin Domain-Containing Protein 3 (NLRP3) inflammasomes as a novel target for regulating extracellular matrix disorganizations and therefore improving therapeutic conditions post surgery [16]. Follow-up studies on rotator cuff tears suggest that stem cells and exosome therapy may be utilized post-op as an aid for rehabilitation [32].

Hyperlipidemia-induced atherosclerosis and inflammation are codetermining variables that promote each other. As xanthomas accumulate within the extracellular matrix of tendons, a macrophagic response is induced [24]. This initial sequestering of oxidized LDL-carriers along with foam cell aggregates within the tendons stimulate Intercellular Adhesion Molecule (ICAM) and Vascular Cell Adhesion Molecule (VCAM) release within endothelial cells further promoting the mobility of neutrophils to the site of hyperlipidemia. Chemokines (CCR2, CCR5, CX3CR1) are released from smooth muscle cells to recruit inflammatory monocytes and T-Cells. Monocytes differentiate into tissue specific macrophages – contributing to plaque buildup [33]. Xanthoma aggregates and fatty deposits within tendons contribute to oxidative damage, obstructed tissue vascularity, persistent inflammatory cytokine production, reduced cholesterol efflux, and matrix turnover by macrophages [34].

### 3.2 Oxidative stress

The impact of hyperlipidemia on rotator cuff tears through the vascular system has been a topic of interest. In hyperlipidemic environments, oxidized LDL lipid carriers can accumulate in the extracellular environment of rotator cuff tendons and cartilage–depositing cholesterol aggregates called xanthomas [1,15,22,24]. The occurrence of these xanthomas is likely due to dietary LDL leaving the circulation and becoming trapped in the dense regular collagen and glycosaminoglycan extracellular matrix of the tendon tissue – then becoming engulfed and oxidized by macrophages. The result is the proliferation of foam cell aggregates (xanthomas) embedded within the rotator cuff tendons which leads to instability [24]. Cholesterol buildup within the foam cells leads to mitochondrial dysfunction, apoptosis, and the eventual necrosis of underlying tissues within the rotator cuff [25]. Chronic hyperlipidemia may cause persistent low-grade inflammation within the tendon, leading to stiffness, matrix degradation, and oxidative stress – ultimately weakening the rotator cuff [16,29]. Precipitating factors like continuous strain, dyslipidemia, sports injury, and trauma may exacerbate the inflammatory pathways involved in rotator cuff tears.

Sustained oxidative stress has been suggested as a factor for tendon fibrosis, adhesion, and scarring following an acute tendon injury. The reactive oxygen species can initiate an inflammatory response and tendon damage if produced at a rate larger than the tendon’s antioxidant capacity. Rotator cuff pathologies have been linked to significantly high expression of S100A11, PLIN4, and HYOU1 – proteins that mediate the inflammatory and anti-hypoxic response. Hyperlipidemia promotes reactive oxygen species to activate the mTOR pathway which leads to fatty infiltration and metaplasia with heterotopic ossification. Hyperlipidemia-induced tendinopathy may be correlated with increased expression of nesfatin-1, an adipokine dysregulated by obesity. Nesfatin-1 suppresses the autophagy-lysosomal pathway used to combat oxidative stress by removing misfolded proteins and organelles. Decreased autophagy further increased mTOR signaling and subsequent tendon disruption. High cholesterol also triggers reactive oxygen species generation, histopathological abnormalities, apoptosis, and autophagy within tendons. The impacts of cholesterol on autophagy and tendinopathy are likely mediated by the reactive-oxygen-species activated AKT/FOXO1 pathway or NF-kB pathway [35]. As mentioned earlier, peritendinous adhesions due to oxidative stress may also lead to exacerbation in inflammation involving tumor necrosis factor (TNF)-α, Interleukin (IL)-1β, Transforming Growth Factor (TGF)-β, Superoxide Dismutase (SOD)1, SOD2, Collagen (COL)1, and Hypoxia Inducible Factor (HIF)-1 α [36]. The role of oxidative stress in tendinopathy is also supported by the fact that Quercetin, an antioxidant, suppressed the expression of Matrix Metalloproteinases (MMPs) and inflammatory mediators, apoptosis, and autophagy in a rat model of tendinopathy [37].

### 3.3 Aging

In humans, there are two different types of skeletal muscle fibers – type I (fast twitch) and type II (slow twitch). As patients age, there is a gradual reduction of skeletal muscle mass throughout the body (sarcopenia) that influences muscle capacity, mobility, and general health within the elderly [38]. While the age-related decline in muscle mass has largely been associated with the decline in total muscle fibers and atrophy of type II fibers, the data presented on the influence of dietary variability and skeletal muscle characteristics is limited [38,39].

Within the four rotator cuff muscles (supraspinatus, infraspinatus, subscapularis, and teres minor), the muscle fibers appear to mostly be type II fibers. Histological analysis of snap frozen infraspinatus muscles within Sprague Dawley Rats reveals the predominance of type II_X_ fibers [40]. In human cadavers of older subjects, the four rotator muscles are comprised of mixed fiber types with the supraspinatus and other external rotators containing mostly slow myosin heavy chains (54%) and the subscapularis/internal rotator being the least slow myosin heavy chain (38%) [41]. Following a rotator cuff tear, the number of type IA (slow) and type IIA (medium) fibers decrease while type IIB (fast) fibers markedly increase [40]. The findings of Gumucio et al. [40], being compared with fiber orientation models of Lovering and Russ would suggest that the external rotator muscles (supraspinatus, infraspinatus, and teres minor) would suffer the worst tissue regeneration following a full-length tear being that they are predominately made of slow twitch fibers. Hyperlipidemia has detrimental effects on the healing of rotator cuff tears, as it increases the stiffness of tendons and the risk of tendon rupture under tension [29].

The effects of age-related muscle degeneration on rotator cuff stability are largely convincing but there is still much to be desired on the role of hyperlipidemia on muscle stability. Some conclusions can however be suggested from the literature. Increased age has been consistently correlated with the prevalence of hypercholesterolemia regardless of socioeconomic status and education [42]. Age relates to hyperlipidemia as the body becomes less able to clear LDL, less able to remove cholesterol through conversion to bile, and reduced activity of the enzyme (cholesterol 7-α hydroxylase) involved in bile acid biosynthesis. Furthermore, an interesting hypothesis states that age-related effects on hyperlipidemia may be modulated by decreased production of growth hormone – an important hormone involved in cholesterol homeostasis [43]. This hypothesis was tested by Parini et al. [44] through the administration of growth hormone in different aged Sprague-Dawley rats to which they found a marked reduction in hypercholesterolemia in the older rats. They concluded that age-dependent hypercholesterolemia can be reduced through growth hormone administration. Age-related increases in hyperlipidemia may influence RCI by disrupting the extracellular matrix through foam cell accumulation and chronic low-grade inflammation inside the rotator cuff [16,25].

RCI remains a very common problem within geriatric populations, with rates as high as 80% in those older than 80 years of age. In addition, RCI in older individuals is less amenable to repair [45]. Increased rates of tendinopathy and tendon rupture are also observed yet the reason remains unclear [46]. Geriatric populations can be particularly susceptible to a loss of functional independence and autonomy because of rotator cuff injury [47]. Age-related loss of tendon cell turnover, proliferation, and stem/progenitor cells can induce a mechanistic reduction in modulus and strength [48]. Macrophage recruitment may also play an important role in skeletal muscle degeneration in older populations by mediating the inflammatory response associated with age and hyperlipidemia, therefore, disturbing tissue regeneration following a full-length rotator cuff tear [12,40]. Furthermore, the incidence of sarcopenia is found to be exacerbated by full-length rotator cuff tears therefore studies that further elucidate the relationship between sarcopenia and hyperlipidemia hold great clinical value [49]. Current management for RCI in elderly populations includes physical therapy, corticosteroid injections, hyaluronate injections, platelet-rich plasma injections, and arthroscopic repair [45,47]. However, there are minimal treatment methods directly addressing hyperlipidemia as a causative agent for RCI in elderly populations. As a result, rotator cuff dysfunction remains a common musculoskeletal problem in the elderly, often underdiagnosed and undertreated [47]. Aging is a risk factor for RCI; while evaluating the risk factors, consideration should be given to differentiate the risk factors between young and elderly [50,51].

### 3.4 Inflammation

As mentioned, hyperlipidemia plays a large role in inflammatory pathways [33]. This can largely be associated with hyperlipidemia-induced chronic low-grade activation of Nuclear-Factor Kappa Beta (NF-κB) followed by canonical activation of proinflammatory factors TNF-α and IL-6 [52,53]. NF-κB has a significant role in the function of the epithelium and skeletal system and is associated with many chronic inflammatory and autoimmune diseases. Downstream activation of NF-κB, TNF-α, IL-1, and Pattern Recognition Receptors (PRRs) within tendon fibroblasts is increased in tendinopathy. Furthermore, the production of IL-6 will stimulate T-cell and macrophage activation with an emphasis on inflammation in the presence of TNF -α. TNF-α and IL-6 stimulate apoptosis of myocytes, catabolism of intramyocellular proteins, and dysregulation of the regeneration pathway [54,55]. In RCI patients, IL-6 gene expression and cytokine production correlate with the extent of degeneration and joint stiffness [56]. One important study by Bhatt et al. [53] determined that hyperlipidemia also decreased IκBα levels in type II muscle fibers but not type I. Within the rotator cuff, these findings may implicate that hyperlipidemia has a disproportionate effect on the subscapularis muscles compared to the external rotators. Further animal studies show an increase in supraspinatus tendon stiffness and decreased tendon elasticity, across many different species, which may also contribute to rotator cuff tears [15,34,57,58]

**Figure 1:**
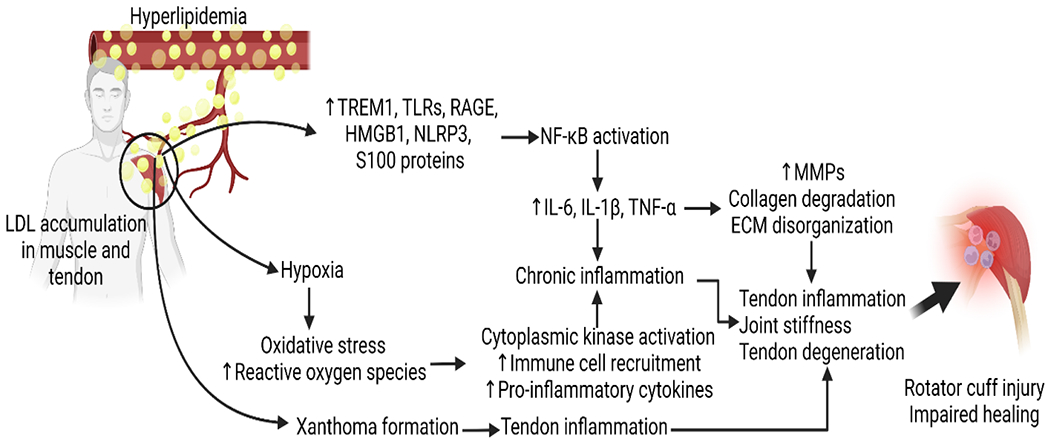
The molecular mechanism of hyperlipidemia-induced rotator cuff injury and impaired repair. Interleukin (IL)–1, Tumor Necrosis Factor (TNF)-α, Matrix Metalloproteinases (MMPs), Extracellular Matrix (ECM), High Mobility Group Box Protein (HMGB)-1, NOD-, LRR- and Pyrin Domain-Containing Protein 3 (NLRP3), Toll-Like Receptor (TLR), Receptor for Advanced Glycation End Products (RAGE), Nuclear Factor Kappa Beta (NF-κB), Triggering Receptor Expressed on Myeloid Cells (TREM)-1.

Detrimental effects of inflammation following rotator cuff tears may be associated with increased Triggering Receptor Expressed on Myeloid Cells (TREM)-1 that is involved in Damage Associated Molecular Proteins (DAMP)-mediated inflammation. In chronically inflamed tendons, TREM-1 may induce macrophage recruitment that leads to ECM disorganization. The ECM provides core support for the essential cell and tissue development. Disorganization within the extracellular matrix following a rotator cuff tear leads to the upregulation of inflammatory cytokines and increased oxidative stress. The inflammatory product of the NLRP3 pathway, IL-1β, induces the degradation of the collagen matrix of tendon and bone connective tissue. Extreme hypoxia following rotator cuff injury can lead to chronic stimulation of the NLRP3 pathway, causing persistent inflammation and ECM degradation within the rotator cuff [16,59]. Mechanical unloading after a tendon tear may also shift muscle cell metabolism from anabolic to catabolic through subsequent mitochondrial dysfunction following a tear. Damage-Associated Molecular Patterns (DAMPs) and pro-inflammatory cytokine recruitment including TNF -α, IL-1, and IL-6 can stimulate catabolic processes within intramyocellular proteins – causing rotator cuff muscle degeneration [54]. The size of the rotator cuff tear is also correlated with the cytokine expression within the synovium of the supraspinatus tendon. Unsurprising, the expression of inflammatory markers consistent with hyperlipidemia (IL-1β, IL-6, COX-2), was found at higher amounts in full-thickness tears compared to partial-thickness tears [60,61]. The upregulation of these genes is consistent with enhanced synovial inflammation, vascular ingrowth, and collagen disorganization across the supraspinatus and subscapularis tendons as determined by histology [60] (Figure 1).

Inflammatory reactions have been linked to rotator cuff impairments through a variety of mechanisms (Figure 1 and Table 1). One such avenue for rotator cuff impairment is inflammation of the subacromial bursa which is thought to cause shoulder pain by stimulating afferent nerve endings [62]. Another study utilized Immunohistochemical techniques to reveal an increase in cytokines and metalloproteases such as IL-1, IL-6, TNF -α, COX-1, and COX-2 in patients with bursitis. COX-1 and COX-2 being cyclooxygenase enzymes can be targeted by NSAID to improve the prognosis of subacromial bursitis [63]. Furthermore, inflammatory reactions can be common following rotator cuff operations with 16% of patients having a local inflammatory response to porcine small intestine submucosa implants – a collagen-based material used in soft tissue repair [12]. microRNA targeting can be a fruitful therapy for controlling the inflammatory response, as miRNA has been associated with key matrix-degrading enzymes such as metalloproteinases [64,65]. While more studies are recommended to further elucidate the relationship between hyperlipidemia and rotator cuff inflammation, the inflammatory response associated with hyperlipidemia likely causes adverse consequences during and after rotator cuff repair.

### 3.5 Hyperlipidemia, bone health, and RCI

Hyperlipidemia can have a profound effect on bone health and regeneration reducing tendon insertion strength and mechanical strength of the rotator cuff [73]. Reports from The National Health and Nutrition Examination Survey suggest that 63% of osteoporotic patients are diagnosed with hyperlipidemia [74,75]. Much like in tendons, hyperlipidemic conditions can cause LDL particles to cross through the endothelial barrier, become oxidatively modified, and become entrapped within the perivascular subendothelial spaces within human osteoporotic bone [75]. Osteoblasts play a role in oxidizing the LDL proteins and increasing the localized number of oxidized products in the bone [13]. Oxidative stress generated by the hyperlipidemic environment has been identified as a marker for rotator cuff pathology as it can impair bone signaling and differentiation and induce an inflammatory response within the muscles [13,14,16,29,34,76]. Reactive oxygen species are found in higher amounts in the synovial fluid of patients with osteoarthritis and rotator cuff tears [35].

Mouse model studies determined to understand the role of oxidized lipids in rotator cuff pathology suggest that hyperlipidemia may induce secondary hyperparathyroidism and impair bone regeneration, stiffness, and mechanical strength within the rotator cuff [13,44]. The effects of hyperlipidemia on peri-implant defect regeneration are even less encouraging – with hyperlipidemia causing a marked decrease in both bone graft regeneration and implant stability in rabbit models [73]. Hyperlipidemia-induced fatty infiltration may weaken the strength of tendons following repair and reduce tendon-to-bone healing of the rotator cuff [49]. Finally, the tendon and bone weakness associated with hyperlipidemia may generate increased mechanical overload of the rotator cuff microenvironment. Mechanical stress on tenocytes can disrupt the tendon microenvironment leading to disrupted ECM organization, collagen composition, and apoptosis [77].

Consequent to the decreased bone regeneration, signaling, stability, strength, and function caused by the hyperlipidemic environment, rotator cuff stability falters. In a cohort study looking at postoperative arthroscopic repair for full-thickness rotator cuff tears, patients with reduced bone mineral density had a significantly larger failure rate of post-operative rotator cuff healing [78]. Likewise in rat models, osteoporosis impairs the healing of rotator cuffs following repair [79]. Hyperlipidemia-induced bone loss at the head of the humerus leads to decreased tendon insertion strength across the infraspinatus enthesis [26]. This may be related to the increased osteoclast migration to the tendon-bone interface at the site of repair [79]. Vitamin D modulation may be of therapeutic benefit to patients who experience osteoporotic-related should pain [21]. These findings demonstrate sound evidence for the detrimental impact of hyperlipidemia on rotator cuff re-tears and healing post operation however more evidence is recommended to solidify the role of osteoclast migration during rotator cuff repair and to better understand hyperlipidemia-induced bone loss contribution to the incidences of initial full-thickness.

## 4. Existing Treatment Strategies

### 4.1 Rotator Cuff Surgery

Rotator cuff pathology constitutes more than 4.5 million physician visits and more than 75,000 rotator cuff surgeries each year; as a result, rotator cuff surgical techniques have rapidly progressed with arthroscopic repairs being most used today [22]. This technique utilizes capsular and coracohumeral ligament release as well as suture anchors to improve prognosis in 85-95% of cases [22,80]. However, tendon-to-bone healing after rotator cuff surgery has a failure rate of 20%-94% [21]. Although arthroscopic intervention improves outcomes, several factors are interfering with healing leading to impaired healing or retear. Hypercholesterolemia, older age, smoking, diabetes, osteoporosis, fatty infiltration in muscle, muscle atrophy, larger tear size, and greater muscle-tendon unit retraction are common factors that influence healing after surgical repair [81]. Out of these factors, the association of aging with dyslipidemia, presence of hyperlipidemia, and fatty infiltration in muscle are correlated to each other while muscle atrophy with aging and osteoporosis are different aspects of aging. This suggests that the effect of aging on surgical outcomes may be due to different factors and hyperlipidemia with aging is an important mediator. This is because, dyslipidemia not only increases inflammation but also is associated with fatty infiltration and muscle wasting [82], the three important factors involved in impaired repair after surgery. Further, hyperlipidemia is also associated with an increased incidence of osteoporosis [83,84], obesity and metabolic syndrome [85], and smoking [86]; other factors involved in impaired repair after surgery. To note, hyperlipidemia is a common mediator among all these, and this indicates that targeting hyperlipidemia before and after rotator cuff tendon repair should be the focus to improve surgical outcomes. This notion is supported by the findings of decreased improvement after treatment for RCI in patients with dyslipidemia (low HDL and high LDL) [87]. Studies reported that the use of statins to lower cholesterol after RCT repair may help in improving outcomes [88] but the occurrence of rhabdomyolysis with the use of statins raises concerns [17,18] and should be investigated. Obesity is another factor that has been reported to be associated with impaired enthesis healing after repair [89] and the association of hyperlipidemia with obesity indicates the significance of targeting hyperlipidemia after RCT repair.

Lately, there has been more push for optimization during rehabilitation with collagen and biological augmentation such as bFGF-loaded electrospun poly fibrous membranes implant to mitigate re-tears post-operation. They work by supporting cell attachment as well as accelerating tendon-bone remodeling/healing [90]. Other follow-up translational studies highlight the importance of the disorganization of extracellular matrix and the utility of inflammasomes as biological control markers to promote optimal tissue regeneration [16]. As orthopedic research progresses towards biological augmentation in rotator cuff tears, relevant research on hyperlipidemia and rotator tissue tears increases in clinical value [91].

### 4.2 Non-Surgical Treatment

Acute symptoms of rotator cuff tear are treated by rest, activity modification, ice application, and physiotherapy. These strategies help to attenuate flare-up of symptoms, and inflammation, settle down the injury, reduce further damage to the rotator cuff, and strengthen the rotator cuff muscle [92]. In addition to these, lowering down hyperlipidemia will help in repair after surgical repair. The common strategies to lower hyperlipidemia are the use of statins, peroxiredoxin 5, and vitamin D.

Hydroxy-methyl-glutaryl-coenzyme-A reductase inhibitors, better known as Statins, are the most widely used medication to treat Hyperlipidemia. Current evidence suggests that statins can be used to decrease the risk of developing rotator cuff tears by reducing inflammation and fibrosis [15]. Statins can enhance tendon healing by stimulating tenocyte proliferation, migration, and adhesion through increased COX-2 activity and PGE2 signaling [15,93]. Statins are also reported to have negative effects on rotator cuff health as they are associated with myalgia, muscle injury, increased creatine kinase, tendinopathy, and certain tendon ruptures [15,94–96]. A study following 77 patients undergoing rotator cuff repair revealed similar rotator cuff tear rates, fatty infiltration, and patient-reported outcomes between hyperlipidemic patients treated with statin and patients not taking statin [97]. Statins have no effect on retears of rotator cuff muscles after surgery [15,34,91]. Furthermore, an increased risk of rotator cuff tears exists in patients with hyperlipidemia with or without statin use and hyperlipidemic patients experience more pain after non-surgical treatment compared to non-hyperlipidemic patients [1]. Finally, when statin was used peri-operatively following full-thickness arthroscopic repairs in patients with dyslipidemia, no difference in functional outcomes was observed [98].

Peroxiredoxin 5, a thioredoxin peroxidase with antioxidant properties, has been found to protect human tendon cells from loss of function and apoptosis during oxidative stress. Inhibition of mTOR with rapamycin treatment has been used to alleviate nesfatin-1 expression and reduce heterotopic ossification within tendons both in vitro and in vivo. N-acetyl Cysteine (NAC)1 and Forkhead Box O1 (FOX01) inhibitors may reduce the harmful impacts of hypercholesterolemia by rescuing apoptosis and autophagy. NAC and BAY11-7082, a IkB-kinase inhibitor, reverse the inhibitory effects of cholesterol by blocking NF-kB activation [35]. Peroxiredoxin 5 directly reduces hydrogen peroxide and neutralizes other reactive oxygen species. The hydrogen peroxide and other reactive oxygen species cause significant tendon swelling, impaired tendon healing, pain, and structural abnormalities [99]. Peroxiredoxin 5 expression may serve as a protective factor from oxidative stress by reducing apoptosis and maintaining collagen synthesis [100].

Vitamin D is a fat-soluble vitamin that has a role in skeletal, immune, and metabolic function contributing to glucose homeostasis, insulin regulation of body weight, and reduction of cardiovascular risk [101]. Modulation of the metabolite Vitamin D (1α,25-dihydroxy vitamin D3) has been shown to successfully influence bone and muscle healing by increasing osteoblast proliferation and differentiation. Vitamin D can improve rotator cuff stability, improving tendon-to-bone healing by reinforcing bone mineral density and strengthening skeletal muscles [21]. Vitamin D can also suppress the reactive-oxygen-species formation and reverse the anti-proliferative and anti-tenogenic effects of dexamethasone on tenocytes [35]. Vitamin D deficiency can lead to oxidative stress, tissue inflammation, and atherosclerosis by upregulating the expression of monocytes and macrophages within vascular cell intima [102]. Vitamin D supplementation has been suggested as an intervention for alleviating atherogenic dyslipidemia in patients with metabolic syndrome as vitamin D insufficiency is related to both risk and severity of the metabolic syndrome. Levels of serum 25-hydroxy vitamin D are correlated with decreased hypertriglyceridemia and increased levels of HDL-cholesterol [101]. Vitamin D supplementation also lowers total cholesterol and LDL-cholesterol [102,103]. Therefore, targeting of vitamin D may have therapeutic potential as a novel treatment strategy for management of rotator cuff pathology by reducing hyperlipidemia induced inflammation and rotator cuff instability, specifically in patients who are vitamin D deficient [21,101–103].

MicroRNAs (miRNA) are key regulators of inflammatory and fibrotic diseases. Chronic rotator cuff tendinopathy can trace back to select candidate miRNAs including miR-18b, miR-19a, miR-19b, miR-25, miR-93, and miR-192. Specifically, miR-25 may be of importance for regulating the inflammatory response of cytokines TNF-α and HMG. The miR-19 may be a promising target as it is an important modulator of the JAK-STAT signaling pathway and local inflammation [64,65,104]. MicroRNA is also a key regulator of hypercholesterolemia; miRNA-30c interacts with untranslated triglyceride transfer proteins to induce the reduction of apolipoprotein B. Furthermore, miRNA-30c reduces hypercholesterolemia and atherosclerosis in mice by reducing lipid synthesis and secretion of apoB lipoproteins (lipoproteins rich in triglycerides) [105]. miRNA-191 has also been characterized among multiple studies as a potential target involved in the pathogenesis of hyperlipidemia [106,107].

## 5. Conclusion

Overall, the literature at present adequately elucidates the deleterious effects of hyperlipidemia in all components of rotator cuff health. While the effects of hyperlipidemia on bone health and inflammation are compelling, the role of hyperlipidemia on sarcopenia and accompanying rotator cuff function is wanting. These studies have much clinical significance as they can improve the postoperative rehabilitation results in elderly patients as well as underscore the importance of treating hyperlipidemia as a preventive measure for rotator cuff pathology. Targeting underlying mechanisms influencing the hyperlipidemia-inflammation axis has large therapeutic value as a novel treatment strategy for the management of rotator cuff pathology.

## Figures and Tables

**Table 1:** The role of inflammation in rotator cuff pathology.

Study	Sample	Aim	Findings
Krieger et al. [54]	Supraspinatus, harvested 7 days after RCI	Quantitative analysis of immune cell subset infiltration of supraspinatus muscle after severe rotator cuff injury	Results indicate a dramatic increase in macrophage, monocyte, and dendritic cell count following RCI
Shindle et al. [60]	Synovium, bursa, supraspinatus tendon, and subscapularis tendon from RCI patients were evaluated for pro-inflammatory cytokine expression and tissue remodeling.	Determine whether tear size correlates with synovial fluid inflammation and tendon degradation.	IL-1β, IL-6, COX-2, MMP-9, and VEGF were found in excess in the synovium of patients with full-thickness tears. Loss of collagen organization was observed.
Thankam et al. [16]	Macroscopic examination of swine tendon tissues characterized for inflammasome activity	Characterize inflammatory response, ECM disorganization, HMGB1 regulation, and NLRP3 inflammasome activation in the injured rotator cuff tendon	HMGB1 and NLRP3 inflammasome upregulation following RCI leads to increased TLR4, TLR2, TREM1, RAGE, ASC, and IL-1β. Results suggest an association between local inflammation and extracellular matrix disorganization
Bhatt et al. [53]	Wistar rats fed a high-fat diet, skeletal muscle characterized for IkBα, phosphor-p38, and p38 MAPK with Western Blot.	Address effects of diet-induced obesity on cytokine (JNK, MAPK, NF-κB) expression in varying skeletal muscle types	Fiber-dependent reduction of Ikbα in hyperlipidemic rats.
Kim et al. [66]	35 patients undergoing arthroscopic release for shoulder stiffness	Compare genetic association of inflammation and fibrosis in RCI in anterior vs. posterior capsule	More fibrogenic processes occur in the anterior capsule compared to the posterior following RCI
Candela et al. [67]	202 patients undergoing arthroscopic repair following varied size full-thickness RCTs.	Evaluate the association between RCT size and long head of bicep pathology	Shoulder long head of bicep tendon pathology is associated with the size of RCT.
Aagaard et al. [68]	A prospective cohort study of 62 elderly patients with trauma-related full-thickness RCT.	Analyze histopathological features in trauma-related RCT and compare them to non-trauma-related tears.	No difference in degenerative changes between trauma and no trauma-related RCT.
Stengaard et al. [69]	45 C57BL/6 mice subject to supraspinatus tear.	Characterize inflammation, degeneration, fatty infiltration, and regeneration in RCT	Supraspinatus tears show severe inflammation, degeneration a fatty infiltration.
Shinohara et al. [70]	16 patients underwent arthroscopic surgery for RCT with a range of motion limitations.	Study effects of AGE on the range of motion in the shoulder in relation to diabetes	The diabetes group has significant AGE and limited range of motion, potentially due to increased oxidative stress.
Asano et al. [71]	33 patients undergoing arthroscopic repair for RCT	Define association of blood flow in anterior humeral circumflex artery with synovial inflammation.	A positive association between peak humeral circumflex artery blood flow and synovial inflammation
Yoshikawa et al. [72]	Twenty patients over 50 years with non-traumatic RCT	Identify the influence of diabetes-induced glycation and oxidative stress in RCT patients	Diabetes-induced hyperglycemia causes increased advanced glycation end-products and receptors, followed by reactive end products and cell apoptosis.

AGE: Advanced Glycation End Products; ASC: Apoptosis-Associated Speck-Like Protein Containing A CARD; COX-2: Cyclooxygenase-2; ECM: Extracellular Matrix; HMGB-1: High Mobility Group Box Protein-1; IL: Interleukin; JNKs: C-Jun N-Terminal Kinases; MAPKs: Mitogen-Activated Protein Kinases; MMPs: Matrix Metalloproteinases; NF-kB: Nuclear Factor Kappa Beta; NLRP3: NOD-, LRR- And Pyrin Domain-Containing Protein 3; RAGE: Receptor For Advanced Glycation End Products; RCI: Rotator Cuff Injury; RCT: Rotator Cuff Tear; TNF-α: Tumor Necrosis Factor-A; TLR: Toll-Like Receptor; VEGF: Vascular Endothelial Growth Factor

## Data Availability

The findings from the cited publications were critically reviewed and the summary of the findings and conclusion are provided in the manuscript. No additional data are included.
